# Supplementary material for: The biochemical mechanism of Rho GTPase membrane binding, activation and retention in activity patterning
Source: EMBO J. 2025 Mar 31;44(9):2620–57. doi: 10.1038/s44318-025-00418-z (PMC12048676; doi:10.1038/s44318-025-00418-z)
Supplement: Supplementary file 1 — Appendix [file 44318_2025_418_MOESM1_ESM.pdf]

Mechanistic principles of Rho GTPase patterning

Michael C. Armstrong, Yannic R. Weiß, Lila E. Hoachlander-Hobby, Ankit A. Roy, Ilaria Visco, Alison Moe, Adriana E. Golding, Scott D. Hansen, William M. Bement*, Peter Bieling*

*Correspondence to: William M. Bement, wmbement@wisc.edu, Peter Bieling, peter.bieling@mpi-dortmund.mpg.de

Content

Appendix Figures

Appendix Figure 1 P2

Appendix Figure 2 P3

Appendix Figure 3 P4

Appendix Figure 4 P6

Appendix Figure 5 P7

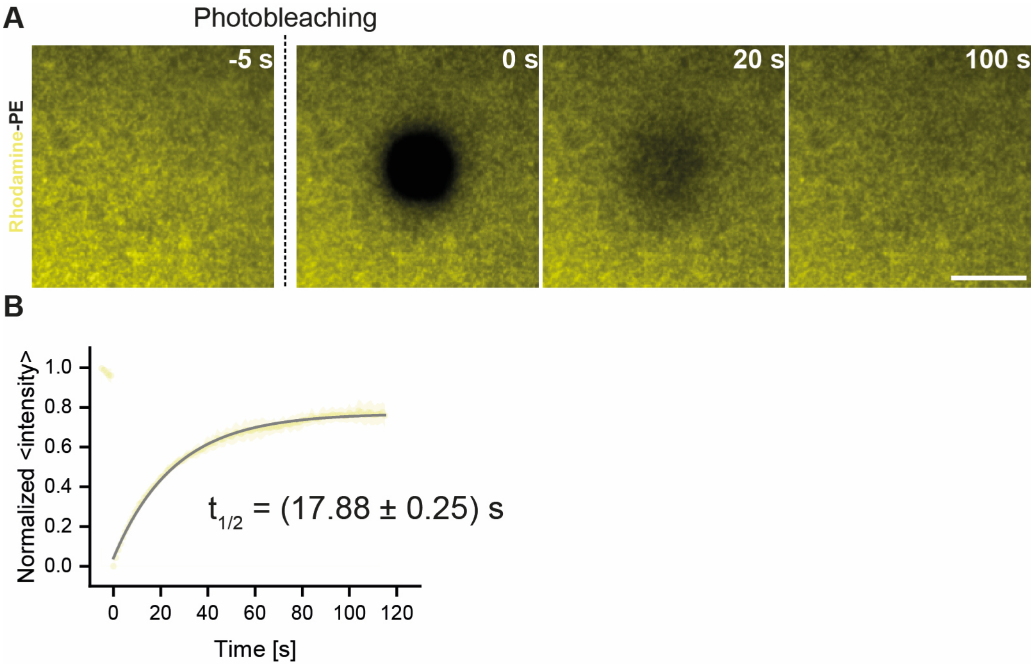

Appendix Figure 1. Fluidity of SLBs made with the PM-Mix, containing PI(4)P and PI(4,5)P_2, determined by fluorescence recovery after photobleaching (FRAP). (**A**) Time-lapse TIRFM images of rhodamine-PE (0.01% in the PM-Mix, yellow) at indicated times before or after photobleaching at t = 0 s. (**B**) Normalized fluorescence intensity in FRAP regions plotted over time and fitted with a mono exponential function to obtain $t_{1/2}$. All numeric data represent the mean from five independent experiments (symbols) ± SD (shaded areas) (N=5). All scale bars are 10 µm.

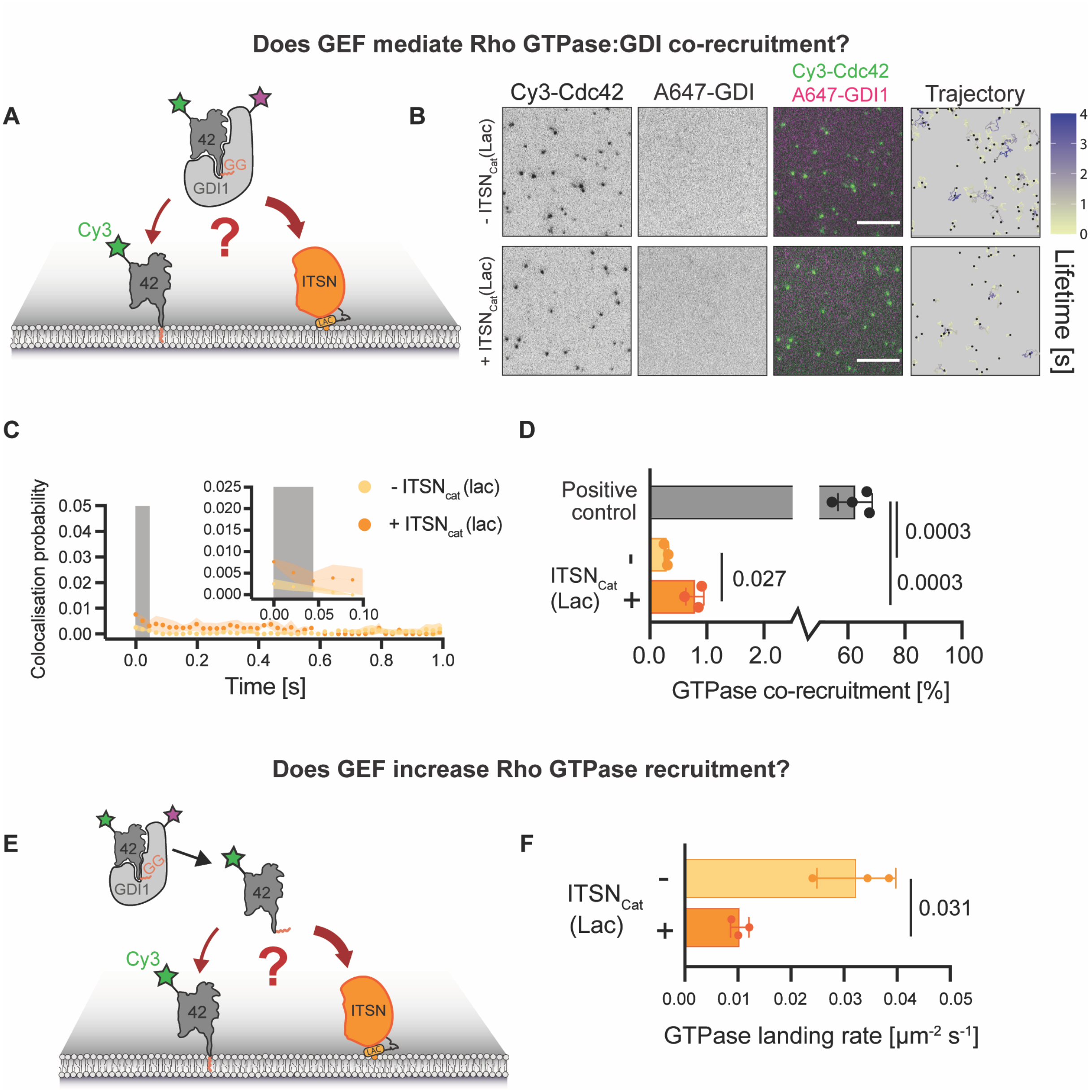

Appendix Figure 2. Attachment chemistry of GEF does not influence Cdc42:GDI1 recruitment. (**A**) Scheme of two potential routes to GEF-mediated GTPase recruitment and activation. (**B**) TIRFM images of single molecule recruitment of Cdc42 from complex (100 pM) (Cy3-Cdc42, A647-GDI1, merge, and trajectories of Cdc42, left-to-right) on a PM SLB in the absence (top) or presence (bottom) of 400 nM $ITSN_{Cat}$(Lac). Scale bar = 10 µm. (Upper images also used in Fig 3G) (**C**) Probability (dots) ± SD (area) of Cy3-Cdc42 and A647-GDI1 co-localization as a function of Cdc42 lifetime on SLBs coated with (orange) or without (yellow) $ITSN_{Cat}$(Lac). t = 0 s is the moment of recruitment. Vertical grey box demarks the frames evaluated for co-recruitment. – $ITSN_{Cat}$(Lac) n = 8226, N = 3, + $ITSN_{Cat}$(Lac) n = 3578 N = 3. (**D**) Mean fraction ± SD of Cy3-Cdc42 molecules co-recruited with A647-RhoGDI1 to PM SLBs in the absence or presence of $ITSN_{Cat}$(Lac) compared to the positive control. (± GEF experiments N=3, control N=4). (**E**) Scheme of GEF-dependent and independent membrane recruitment of free RhoGTPases. (**F**) Cy3-Cdc42 landing rates (mean ± SD) on PM SLBs in in the absence (orange) or presence (yellow) of $ITSN_{Cat}$(Lac). – $ITSN_{Cat}$(Lac) n = 8226, N = 3, + $ITSN_{Cat}$(Lac) n = 3578 N = 3.

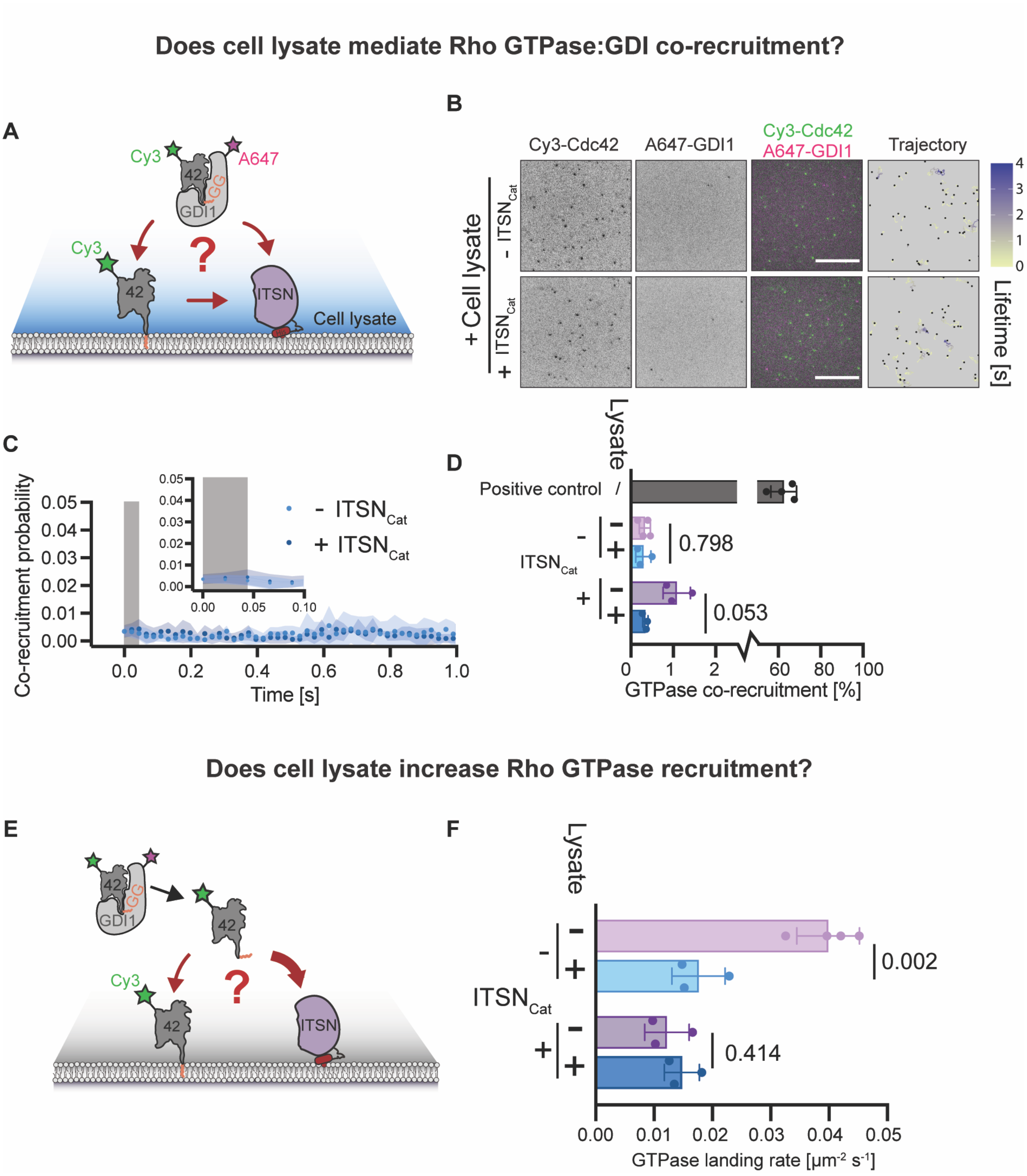

Appendix Figure 3. Cell lysate does not increase Cdc42 recruitment rate. (**A**) Scheme of two potential routes to GEF-mediated GTPase recruitment and activation in the presence of cell lysate from pig brain. (**B**) TIRFM images of single molecule recruitment of Cdc42 from complex (100 pM) (Cy3-Cdc42, A647-GDI1, merge, and trajectories of Cdc42, left-to-right) on a PM SLB (0.25% Ni^{2+}-NTA-DGS)) in the absence (top) or presence (bottom) of 400 nM $ITSN_{cat}$. Experiments completed in cell lysate from big brain. Scale bar = 20 µm. (**C**) Probability (dots) ± SD (area) of Cy3-Cdc42 and A647-GDI1 co-localization as a function of Cdc42 lifetime on SLBs coated with (dark blue) or without (light blue) $ITSN_{Cat}$, in the presence of cell lysate t = 0 s is the moment of recruitment. Vertical grey box demarks the frames evaluated for co-recruitment. – $ITSN_{cat}$. N = 3, n = 4284, + $ITSN_{cat}$ N = 3, n = 3599. (**D**) Mean fraction ± SD of Cy3-Cdc42 molecules co-recruited with A647-RhoGDI1 to PM SLBs in the absence or presence of $ITSN_{Cat}$ compared to the positive control in the presence or absence of

cell lysate (± GEF experiments N=3, control N=4). (± GEF experiments N=3, control N=4). (**E**) Scheme of GEF-dependent and independent membrane recruitment of free RhoGTPases. (**F**) Cy3-Cdc42 landing rates (mean ± SD) on PM SLBs in the presence or absence of $ITSN_{cat}$. – $ITSN_{cat}$ and presence and absence of cell lysate. (- Lysate - $ITSN_{cat}$ N =3, n = 6106. - Lysate + $ITSN_{cat}$ N = 3, n = 5944. + Lysate - $ITSN_{cat}$ N=3, n = 4284. + Lysate + $ITSN_{cat}$ N = 3, n = 3599).

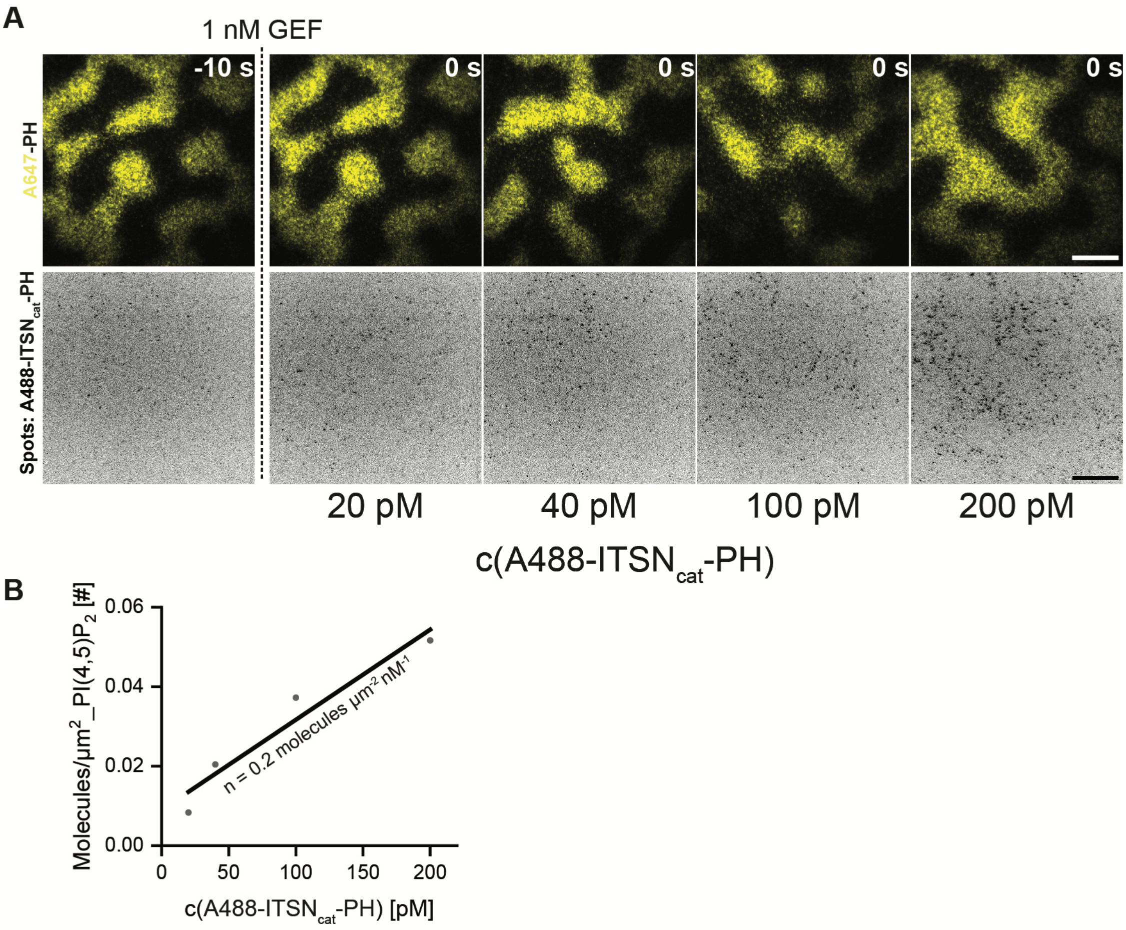

Appendix Figure 4. **Density of ITSN$_{cat}$-PH in PI(4,5)P$_2$ areas.** (**A**) TIRFM images of A647-PH (2 nM, top) and single molecules of A488-ITSN$_{cat}$-PH (varying concentration, bottom) at indicated times before and after addition of (A488)-ITSN$_{cat}$-PH (Total concentration: 1 nM) at t = 0 s. (**B**) Number of molecules per µm^2 in regions of high PI(4,5)P$_2$ plotted against the corresponding concentration of labelled GEF and fitted with a linear function to obtain the density of GEFs inside regions of high PI(4,5)P$_2$.

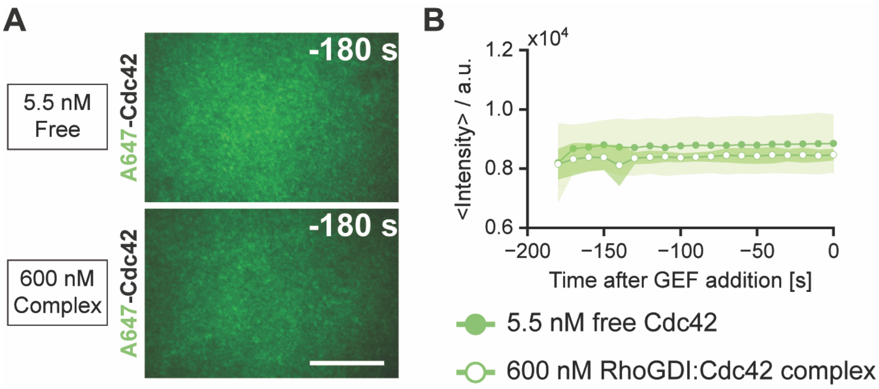

Appendix Figure 5. **Matching membrane loading of free Cdc42 and in complex with RhoGDI1.** (**A**) TIRFM images of A647-Cdc42 (5.5 nM, top) and A647-Cdc42:RhoGDI1 complexes (600 nM, bottom) on PIP patterns before addition of ITSN$_{cat}$-PH at t = 0 s. (**B**) Average intensities of A647-Cdc42 (filled circles) and A647-Cdc42:RhoGDI1 complexes (hollow circles) on the membrane over time. All numeric data represent the mean from three independent experiments (symbols) ± SD (shaded areas) (N=3). All scale bars are 20 µm as indicated. (Image from **A (**upper) also used in Fig 5J) and (lower) also used in Fig 6B (left) and Figure EV4D (left).
