# Supplementary material for: The biochemical mechanism of Rho GTPase membrane binding, activation and retention in activity patterning
Source: EMBO J. 2025 Mar 31;44(9):2620–57. doi: 10.1038/s44318-025-00418-z (PMC12048676; doi:10.1038/s44318-025-00418-z)
Supplement: Supplementary file 3 — Movie EV 1 [file 44318_2025_418_MOESM3_ESM.zip › EMBOJ-2024-119022R-Movie_EV_1.docx]

**Movie EV1. Enrichment of IT-Cdc42 and wGBD around single cell wound.** Confocal 4D time lapse movie of mCh-wGBD (left) IT-GFP-Cdc42 (center) and merge (right). Maximum intensity projection of 5-stack movie (1μm step size; time interval = 9.8 sec; Scale bar = 20 µm). Corresponding to Figure 1B.
