# Supplementary material for: The biochemical mechanism of Rho GTPase membrane binding, activation and retention in activity patterning
Source: EMBO J. 2025 Mar 31;44(9):2620–57. doi: 10.1038/s44318-025-00418-z (PMC12048676; doi:10.1038/s44318-025-00418-z)
Supplement: Supplementary file 4 — Movie EV 2 [file 44318_2025_418_MOESM4_ESM.zip › EMBOJ-2024-119022R-Movie_EV_2.docx]

**Movie EV2**. **Single molecule recruitment of Cdc42 from Cdc42:GDI1 complex to supported lipid bilayer in the absence or presence of ITSN_cat_**. TIRFM movie of the recruitment of Cy3-Cdc42 (left), AF647-RhoGDI1 (center) and merge (right) to a plasma membrane-mimicking supported lipid bilayer containing 2% Ni-NTA in the absence (upper) or presence (lower) of GEF. 100 pM of GTPase:GDI complex in solution was added in the moments before t = 0. Continuous simultaneous imaging (22 ms exposure) Scale bar = 10 µm. Corresponding to Figure 3G.
