# Supplementary material for: The biochemical mechanism of Rho GTPase membrane binding, activation and retention in activity patterning
Source: EMBO J. 2025 Mar 31;44(9):2620–57. doi: 10.1038/s44318-025-00418-z (PMC12048676; doi:10.1038/s44318-025-00418-z)
Supplement: Supplementary file 5 — Movie EV 3 [file 44318_2025_418_MOESM5_ESM.zip › EMBOJ-2024-119022R-Movie_EV_3.docx]

**Movie EV3. GEF localisation on self-organizing PI4P and PI(4,5)P_2_ lipids.** Multi-color TIRFM movie of PIP lipid patterns on PM SLBs visualized by A647-PH (2 nM, yellow) at indicated times before or after addition of A488-ITSN_cat_-PH (1 nM, purple) at *t* = 0 s. Corresponding to Figure 4B,D.
