# Supplementary material for: The biochemical mechanism of Rho GTPase membrane binding, activation and retention in activity patterning
Source: EMBO J. 2025 Mar 31;44(9):2620–57. doi: 10.1038/s44318-025-00418-z (PMC12048676; doi:10.1038/s44318-025-00418-z)
Supplement: Supplementary file 6 — Movie EV 4 [file 44318_2025_418_MOESM6_ESM.zip › EMBOJ-2024-119022R-Movie_EV_4.docx]

**Movie EV4. Local activation of Cdc42 in the absence or presence of OPHN1_cat_.** Multi-color TIRFM movie of Cy3-PH (2 nM, yellow), A488-wCRIB (40 nM, magenta) and A647-Cdc42 (5.5 nM, green) on PIP patterns at indicated times before or after addition of ITSN_cat_-PH (1 nM) at *t* = 0 s in the absence (left) or presence (right) of OPHN1_cat_ (20 nM). Corresponding to Figure 4F,J.
