# Supplementary material for: The biochemical mechanism of Rho GTPase membrane binding, activation and retention in activity patterning
Source: EMBO J. 2025 Mar 31;44(9):2620–57. doi: 10.1038/s44318-025-00418-z (PMC12048676; doi:10.1038/s44318-025-00418-z)
Supplement: Supplementary file 7 — Movie EV 5 [file 44318_2025_418_MOESM7_ESM.zip › EMBOJ-2024-119022R-Movie_EV_5.docx]

**Movie EV5. Effects of RhoGDI1, full length N-WASP or 2xwCRIB on stable Cdc42 activity patterns.** Multi-color TIRFM movie of the effects of RhoGDI1, full length N-WASP or 2xwCRIB on templated RhoGTPase activity patterns. Left: Movie of Cy3-PH (2 nM, yellow), A488-wCRIB (40 nM, magenta) and A647-Cdc42:RhoGDI1 complexes (600 nM, green). Middle: Movie of Cy3-PH (2 nM, yellow), Atto488-N-WASP (40 nM, magenta) and A647-Cdc42 (5.5 nM, green). Right: Movie of Cy3-PH (2 nM, yellow), A488-2xwCRIB (20 nM, magenta) and A647-Cdc42 (5.5 nM, green). All on PIP patterns at indicated times before or after addition of ITSN_cat_-PH (1 nM) at *t* = 0 s in the presence of OPHN1_cat_ (20 nM). Corresponding to Figure 5B,E,H.
