# Supplementary material for: The biochemical mechanism of Rho GTPase membrane binding, activation and retention in activity patterning
Source: EMBO J. 2025 Mar 31;44(9):2620–57. doi: 10.1038/s44318-025-00418-z (PMC12048676; doi:10.1038/s44318-025-00418-z)
Supplement: Supplementary file 8 — Movie EV 6 [file 44318_2025_418_MOESM8_ESM.zip › EMBOJ-2024-119022R-Movie_EV_6.docx]

**Movie EV6. Stability of Cdc42 on membranes in different PIP regions in the presence of wCRIB or 2xwCRIB during continuous flow.** Multi-color TIRFM video of PIP-templated Cdc42 activity patterns in the presence of A488-wCRIB (left) or A488-2xwCRIB (right) at indicated times before or after buffer flow-out at t = 0 s. Flow-out buffer contained all proteins at the same concentrations before flow onset, except for A647-Cdc42 that was omitted, and additional free RhoGDI1 (22 nM) to accelerate Cdc42 membrane dissociation. Corresponding to Figure 6F.
