# Supplementary material for: The biochemical mechanism of Rho GTPase membrane binding, activation and retention in activity patterning
Source: EMBO J. 2025 Mar 31;44(9):2620–57. doi: 10.1038/s44318-025-00418-z (PMC12048676; doi:10.1038/s44318-025-00418-z)
Supplement: Supplementary file 9 — Movie EV 7 [file 44318_2025_418_MOESM9_ESM.zip › EMBOJ-2024-119022R-Movie_EV_7.docx]

**Movie EV7. Photoactivation of Cdc42 before wounding, in the Cdc42 zone following wounding, and outside the Cdc42 zone.** Confocal 3D time lapse movie of mCh-wGBD (left) IT-PAGFP-Cdc42 (center) and merge (right). Single focal plane movie (time interval = 0.43 sec. Scale bar = 20 µm. 405 nm laser used to photoactivate. Corresponding to Figure 7B, E, H.
