# Supplementary material for: The biochemical mechanism of Rho GTPase membrane binding, activation and retention in activity patterning
Source: EMBO J. 2025 Mar 31;44(9):2620–57. doi: 10.1038/s44318-025-00418-z (PMC12048676; doi:10.1038/s44318-025-00418-z)
Supplement: Supplementary file 10 — Expanded View Figures [file 44318_2025_418_MOESM10_ESM.pdf]

Expanded View Figures

Figure EV1. Cy3-Cdc42 follows same patterning as IT-Cdc42 around wounds.

(**A**) Micrograph of Cy3-Cdc42 during Pre-wound, Pre-zone, and Zone time points in the repair process. Scale bar 20 µm. (**B**) Kymograph of the micrograph from (**A**) generated by radially averaging signal intensity around the wound over time (Moe et al, 2021; see "Methods"). The arrow denotes when the wound occurred, and W denotes the wound location. The yellow line "P" indicates the Pre-zone time point and the yellow line "Z" indicates the Zone time point. Vertical and horizontal scale bars are 30 s and 3 µm, respectively. (**C**) Line scan generated from the kymograph in (**B**) to show Cy3-Cdc42 fluorescence intensity at the Pre-zone time point as a function of distance from the wound center. (**D**) Line scan generated from the kymograph in (**B**) to show Cy3-Cdc42 fluorescence intensity at the Zone time point as a function of distance from the wound center. (**E**) Patterning index of Cy3-Cdc42 at the Pre-zone time point compared to the Zone time point. A two-sample t-test was used to determine statistical significance; $n = 13$. P value = 6.48965E-07. (**F**) Patterning index of Cy3-Cdc42 compared to IT-Cdc42 (IT-Cdc42 data were taken from Fig. 1). A two-sample t-test was used to determine statistical significance. Cy3-Cdc42 $n = 13$ individual cells, IT-Cdc42 $n = 12$ individual cells, in $N = 1$ experiment. (**G**) Example of wGBD and IT-Cdc42 timing in cells where both accumulate rapidly. (**H**) Example of wGBD and IT-Cdc42 timing in cells where both accumulate more slowly. (**I**) Timing of IT-RhoA and rGBD enrichment around the wound (mean, $N = 11$ per condition, shaded area = SD).

 © The Author(s)

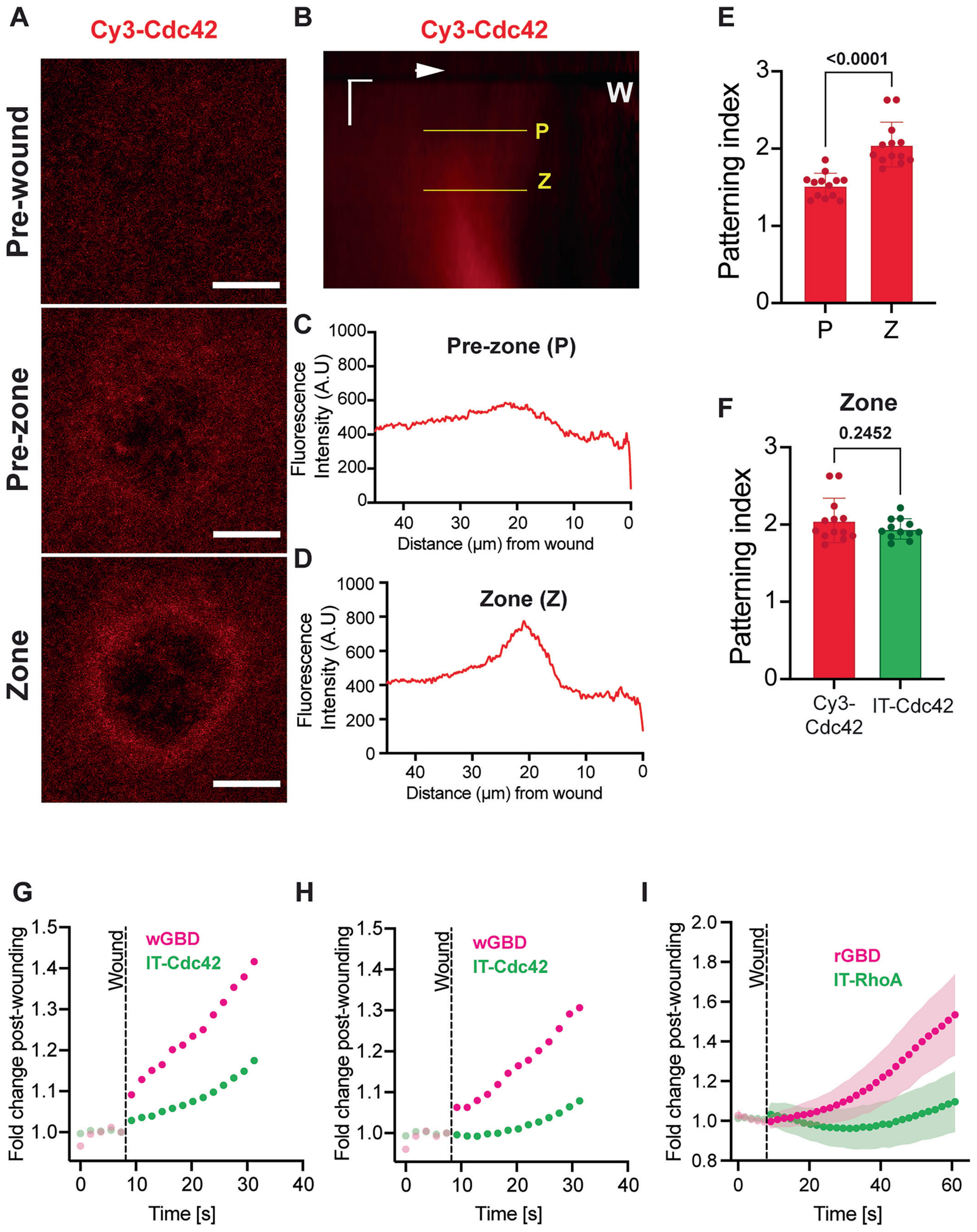

A
Cy3-Cdc42
Pre-wound
Pre-zone
Zone
B
Cy3-Cdc42
W
P
Z
C
Pre-zone (P)
Fluorescence Intensity (A.U)
Distance (µm) from wound
D
Zone (Z)
Fluorescence Intensity (A.U)
Distance (µm) from wound
E
Patterning index
<0.0001
P
Z
F
Zone
0.2452
Patterning index
Cy3-Cdc42
IT-Cdc42
G
Fold change post-wounding
Wound
wGBD
IT-Cdc42
Time [s]
H
Fold change post-wounding
Wound
wGBD
IT-Cdc42
Time [s]
I
Fold change post-wounding
Wound
rGBD
IT-RhoA
Time [s]

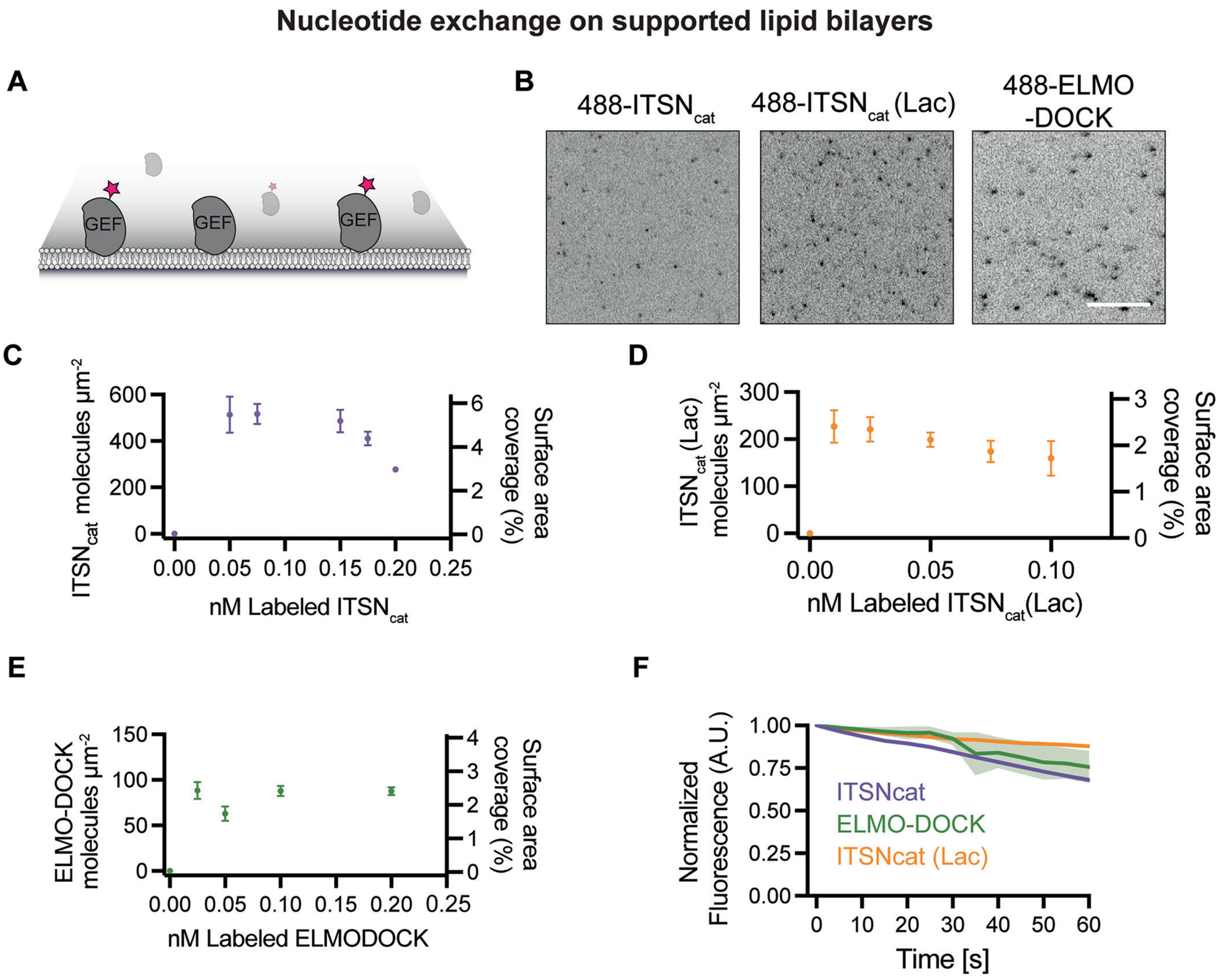

Figure EV2. Achieving bilayer surface coverage of active GEF.

(**A**) Scheme of the single molecule quantification of RhoGEF density on SLBs. (**B**) 488-ITSN$_{cat}$ (left) 488-ITSN$_{cat}$(Lac) (center) and 488-ELMO:DOCK (right) recruited to SLBs (containing 2% DGS-NTA(Ni), 22.5% PS and 2% PI(3,4,5)P$_3$ respectively) Scale bar = 10 µm. (**C**) Quantification of ITSN$_{cat}$ density on SLBs (454 molecules/µm^2, 4.8% surface coverage), left axis. Estimated bilayer surface area coverage of total ITSN$_{cat}$ right axis (mean ± SD). $N = 1$ experiment, $n = 3$–13 fields of view imaged per condition, total spots = 49823. (**D**) Quantification of ITSN$_{cat}$(Lac) density on SLBs. (203 molecules/µm^2, 2.2% surface coverage), left axis. Estimated bilayer surface area coverage of total ITSN$_{cat}$(Lac) right axis (mean ± SD) $N = 1$ experiment, $n = 3$–15 fields of view imaged per condition, total spots = 34044. (**E**) Quantification of ELMO-DOCK density on SLBs. (84 molecules/µm^2, 2.3% surface coverage), left axis. Estimated bilayer surface area coverage of total ELMO-DOCK right axis (mean ± SD) $N = 1$ experiment, $n = 2$–11 fields of view imaged per condition, total spots = 17985. (**F**) Survival fraction of 400 nM 488-ITSN$_{cat}$, 400 nM 488-ITSN$_{cat}$(Lac) and 80 nM 488-ELMO-DOCK as a function of time during wash off (shaded area, SD). Flow rate 10 µL/s.

 © The Author(s)

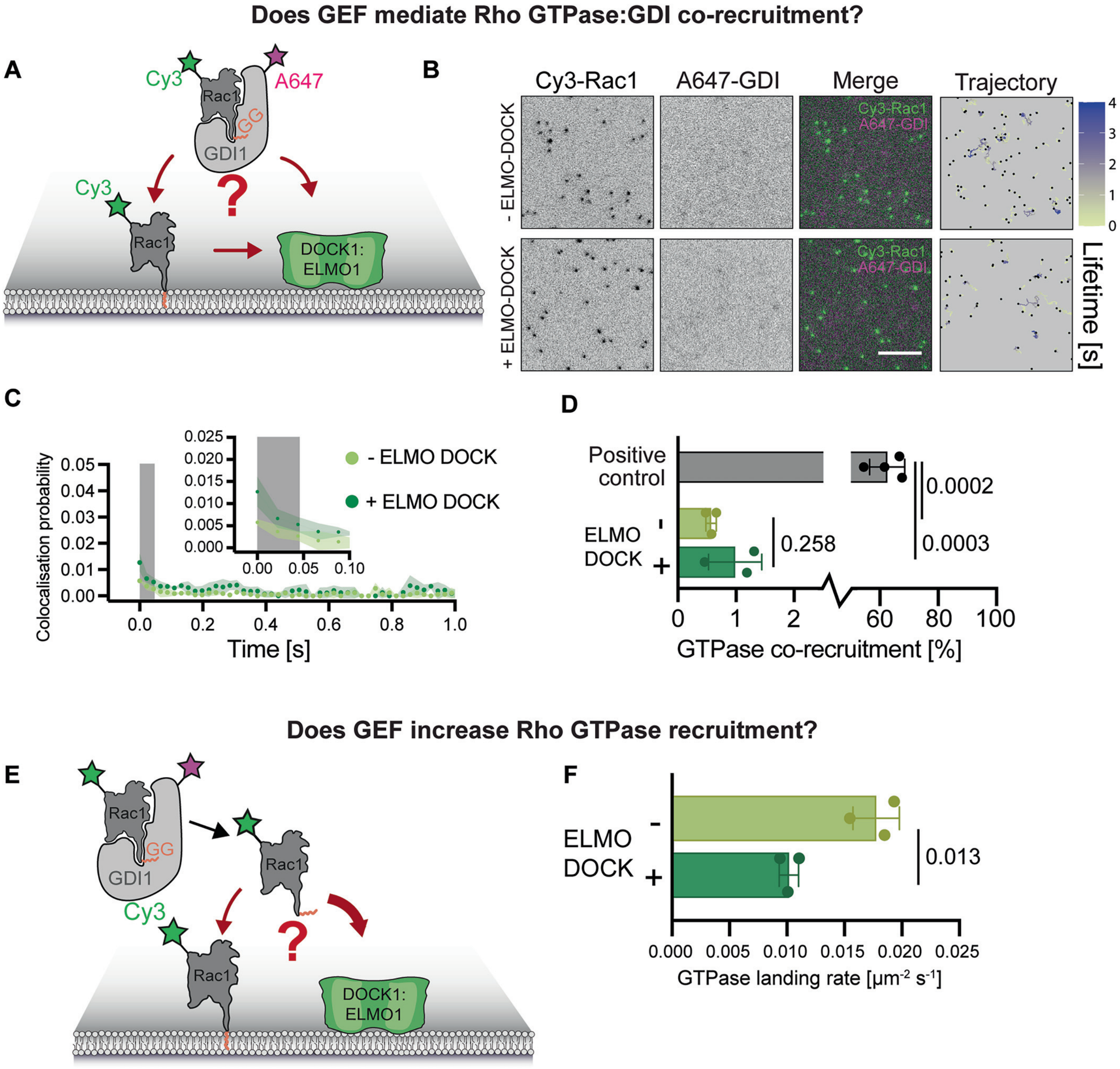

Figure EV3. DOCK family GEF does not provide mechanism for Cdc42:GDI1 recruitment.

(**A**) Scheme of two potential routes to GEF-mediated GTPase recruitment and activation. (**B**) TIRFM images of single molecule recruitment of Rac1 from complex (100 pM) (Cy3-Rac1, A647-GDI1, merge, and trajectories of Cdc42, left-to-right) on a PM SLB (containing 4% PI(3,4,5)P_3) in the absence (top) or presence (bottom) of 80 nM ELMO-DOCK. Scale bar = 10 µm. (**C**) Probability (dots) ± SD (area) of Cy3-Rac1 and A647-GDI1 co-localization as a function of Rac1 lifetime on SLBs coated with (dark green) or without (light green) ELMO-DOCK. $t = 0$ s is the moment of recruitment. Vertical grey box demarks the frames evaluated for co-recruitment. – ELMO-DOCK $N = 3$, $n = 5068$, + ELMO-DOCK $N = 3$, $n = 2984$. (**D**) Mean fraction ± SD of Cy3-Rac1 molecules co-recruited with A647-RhoGDI1 to PM SLBs in the absence or presence of ELMO-DOCK compared to the positive control (Fig. 3E). (± GEF experiments (– ELMO-DOCK $N = 3$, $n = 5068$, + ELMO-DOCK $N = 3$ $n = 2984$, control $N = 4$ $n = 968$, SD)). Statistical comparisons via T-test. (**E**) Scheme of GEF-dependent and independent membrane recruitment of free Rho GTPases. (**F**) Cy3-Rac1 landing rates (mean ± SD) on PM SLBs in in the absence (light green) or presence (dark green) of ELMO-DOCK. – ELMO-DOCK $N = 3$ independent experiments, $n = 5068$ observations, + ELMO-DOCK $N = 3$ independent experiments, $n = 2984$ observations. Statistical comparisons via t-test.

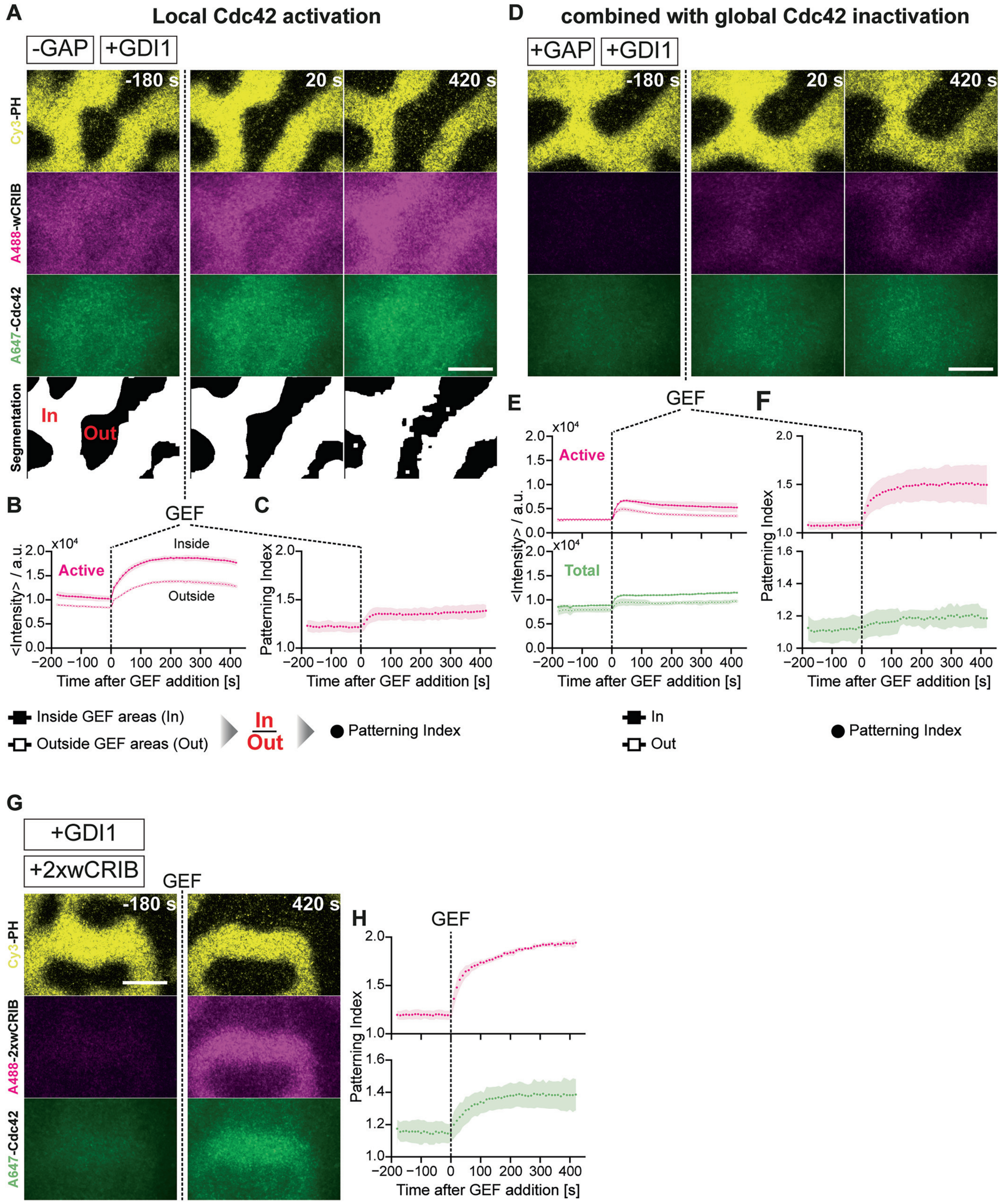
A
Local Cdc42 activation
-GAP
+GDI1
-180 s
20 s
420 s
Cy3-PH
A488-wCRIB
A647-Cdc42
Segmentation
In
Out
B
GEF
C
Active
Inside
Outside
<Intensity> / a.u.
x10⁴
Patterning Index
Time after GEF addition [s]
Inside GEF areas (In)
Outside GEF areas (Out)
In/Out
Patterning Index
D
combined with global Cdc42 inactivation
+GAP
+GDI1
E
F
Active
Total
In
Out
G
+GDI1
+2xwCRIB
A488-2xwCRIB
H

 © The Author(s)

Figure EV4. RhoGDI1 negligibly affects the reconstitution of membrane-templated Rho GTPase activity patterns in vitro.

(**A**) Time-lapse multi-color TIRFM images of Cy3-PH (2 nM, yellow), A488-wCRIB (40 nM, magenta) and A647-Cdc42:RhoGDI1 complexes (600 nM, green) on PIP patterns at indicated times before or after addition of $ITSN_{cat}$-PH (1 nM) at $t = 0$ s. Bottom row shows segmentation based on the lipid pattern. (**B**) Average intensities inside (filled squares, $<I_{In}>$) and outside (hollow squares, $<I_{Out}>$) of the GEF-containing membrane areas or (**C**) patterning index (full circle, $PI = <I_{In}> / <I_{Out}>$) of A488-wCRIB (magenta) over time. (**D–F**) correspond to (**A–C**), with the additional presence of $OPHN1_{cat}$ (20 nM). However, the segmentation is not shown in (**D**) (Image from (**D**) also used in Fig. 6B and Appendix Fig. S5A (lower). Additionally, in (**E**) are the average intensities inside and outside of the GEF-containing membrane areas and in (**F**) the patterning index of A647-Cdc42 (green) over time. (**G**) Time-lapse multi-color TIRFM images of the effects of the synthetic dimerization mimic 2xwCRIB on templated Rho GTPase activity patterns. Conditions as in (**D**) with A488-wCRIB replaced by A488-2xwCRIB (20 nM, magenta). (**H**) corresponds to (**F**) under conditions as in (**G**). All numeric data represent the mean from three independent experiments (symbols) ± SD (shaded areas) ($N = 3$). All scale bars are 20 µm as indicated.

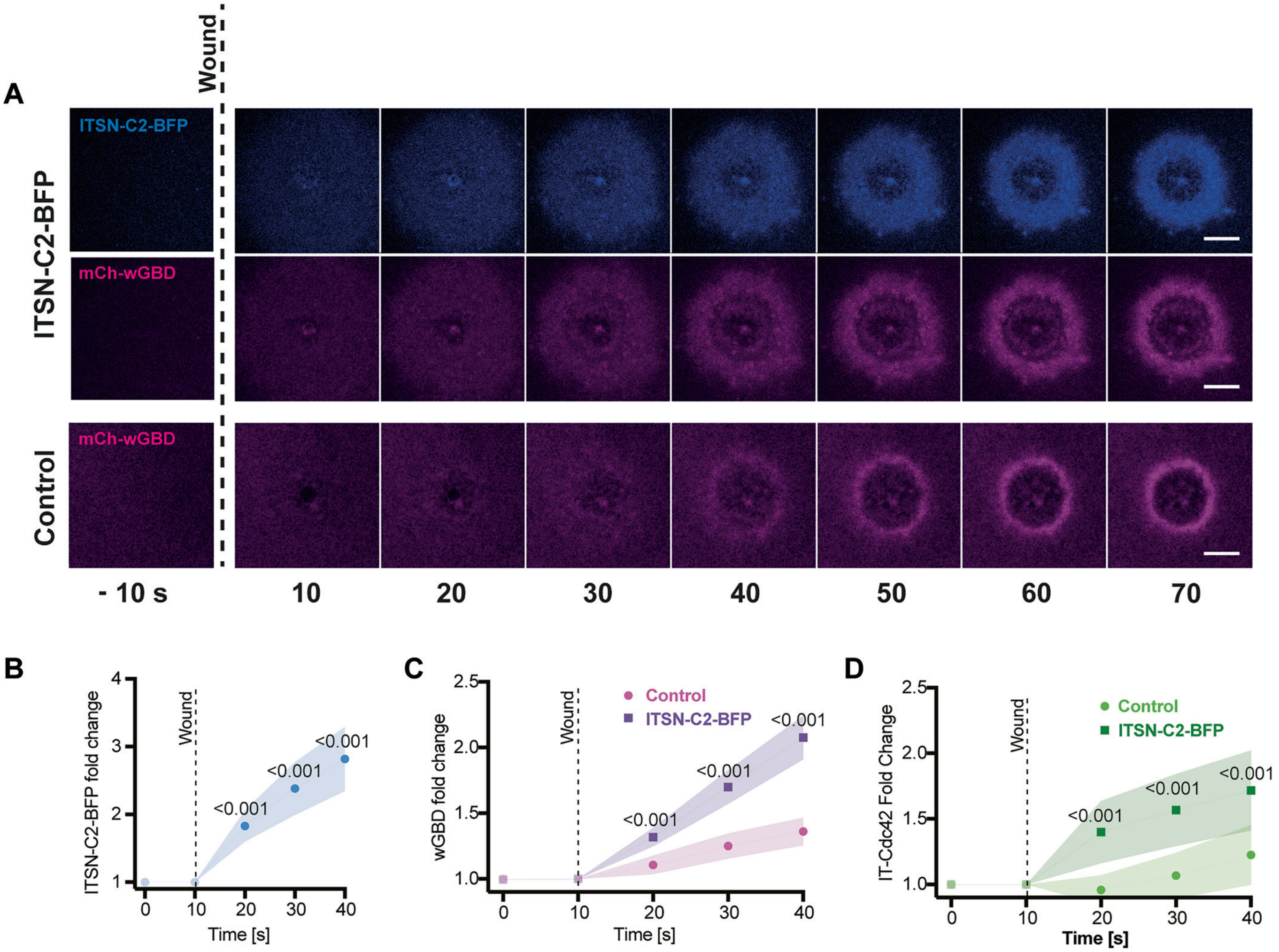

Figure EV5. ITSN-C2-BFP is recruited to single cell wounds and increases Cdc42 activity and patterning.

(**A**) Cell expressing Itsn-C2-BFP (Itsn-C2-BFP) or not (Control). Itsn-C2-BFP Top: Montage of images from confocal time-lapse movie showing accumulation of ITSN-C2-BFP around wounds. Itsn-C2-BFP Bottom: corresponding wGBD signal from images shown in top. Control: example of wGBD signal in cell not expressing ITSN-C2-BFP for comparison. Time in min:sec. Scale bar 20 µm. (**B**) Plot of Itsn-C2-BFP over time. Data show mean ± SD from 22 cells; relative to pre-wound signal (students t-test, *P* values 4.71E-11, 1.09-10 and 3.75E-16 for timepoints 20, 30 and 40 s respectively). (**C**) Plot comparing wGBD accumulation around wounds over time in control cells and cells expressing Itsn-C2-BFP. Data show mean ± SD from 17 cells; Itsn-C2-BFP versus control (students *t* test, (students t-test, *P* values 5.61E-10, 4.82-13 and 3.91E-16 for timepoints 20, 30 and 40 s respectively). (**D**) Plot comparing IT-Cdc42 accumulation around wounds over time in control cells and cells expressing Itsn-C2-BFP. Data show mean ± SD from 12 cells; Itsn-C2-BFP versus control (students t-test, (students t-test, *P* values 3.63E-4, 6.66E-6 and 2.11E-5 for timepoints 20, 30 and 40 s, respectively).

 © The Author(s)
